# Lower Urinary Tract Symptoms in Male Patients With Multiple Sclerosis: Prevalence and Associations With Quality of Life, Depression, and Anxiety

**DOI:** 10.1002/nau.70118

**Published:** 2025-07-15

**Authors:** Yesim Akkoc, Bedriye Karaman, Asli Koskderelioglu, Ozgul Ekmekci, Neslihan Eskut, Nur Yuceyar

**Affiliations:** ^1^ Department of Physical Medicine and Rehabilitation Ege University Faculty of Medicine Izmir Turkey; ^2^ Department of Neurology Ege University Faculty of Medicine Izmir Turkey; ^3^ Izmir Bozyaka Education and Research Hospital, Department of Neurology University of Health Sciences Izmir Turkey

**Keywords:** anxiety, depression, lower urinary tract symptoms, multiple sclerosis, quality of life

## Abstract

**Purpose:**

Lower urinary tract symptoms (LUTS) are frequent in male patients with multiple sclerosis (MS). This study was planned to investigate the frequency and distress of LUTS in men with MS and their association with quality of life (QoL), depression, and anxiety.

**Methods:**

One hundred male patients with MS were included in the study. The International Incontinence Consultation Questionnaire‐Male LUTS questionnaire (ICIQ‐MLUTS) was used to investigate LUTS. The King's Health questionnaire (KHQ) was used to evaluate QoL and the Hospital Anxiety and Depression (HAD‐A, HAD‐D) scale was used to evaluate depression and anxiety.

**Results:**

A total of 100 men with an average age of 39.3 ± 9.3 (range, 20−63) years were evaluated. The mean disease duration was 9.3 ± 6.0 (range, 1−26) years. The mean EDSS score was 2.2 ± 1.8 (median: 2, range: 0−6.5). A total of 91% of patients reported at least one LUTS. The most common storage symptoms were urgency and urgency urinary incontinence (IU), and the most common voiding symptoms were incomplete emptying, hesitancy, and intermittency. ICIQ‐MLUTS incontinence and voiding scores were significantly correlated with disease duration, EDSS, HAD‐A, HAD‐D, and all KHQ subgroups, except general health perceptions.

**Conclusions:**

LUTS, including storage and voiding symptoms, were highly prevalent in male MS patients and significantly correlated with reduced QoL, increased HAD‐D and HAD‐A scores.

## 1 Introduction

Multiple sclerosis (MS) is a chronic inflammatory and degenerative disease of the central nervous system (CNS) that results in demyelination, axonal injury, and neuronal loss. MS involves almost all parts of the CNS, but the spinal cord is one of the most commonly affected areas, serving as a major source of neurogenic lower urinary tract dysfunction (LUTD). Patients with LUTD may experience varying troublesome lower urinary tract symptoms (LUTS) [1], depending on the distribution of MS lesions in the neuroaxis [2]. Some affected individuals present with dominant storage LUTS, such as urinary urgency, increased daytime urinary frequency, nocturia, and urgency IU, wheresa others may experience voiding LUTS, including a slow stream, intermittency, straining to void, and a feeling of incomplete emptying after micturition [3]. Patients with MS with LUTD reported that LUTS limited their daily activities. The physical and clinical burden associated with MS negatively impacted patients' quality of life (QoL) [4], leading to embarrassment, depression, skin fragility, and social isolation [5]. A systematic review showed that LUTS were prevalent in people with MS (68.41%), with frequency (73.45%) and urgency (63.87%) being the most common symptoms [1]. Mahajan et al. found that 65% of 9702 patients reported at least one moderate‐to‐severe urinary symptom. Nocturia and leakage were more common in women, and urgency was more common in men [6].

The questionnaires used to evaluate LUTS vary in studies conducted on patients with MS [4, 6, 7, 8].

LUTS can significantly impact QoL. In the study performed by Tome et al., LUTS affected the QoL of male patients mildly in 43.9%, moderately in 9.8%, and severely in 19.5% [9] A thorough understanding of the nature of LUTS in men with MS can enable individualized treatment strategies, such as lifestyle modifications, medication, and pelvic floor exercises, based on a comprehensive understanding of the patient's specific LUTS profile. This could ultimately lead to improved symptom management and better QoL for these patients.

Given the limited research specifically on LUTS in men with MS, we conducted a cross‐sectional study to determine the prevalence and burden of LUTS in this population. Additionally, we examined the correlation between LUTS and with QoL, depression, and anxiety.

## 2 Materials and Methods

This cross‐sectional study included 100 consecutive male patients with MS recruited from two neurology clinics, one in a university hospital and the other in a state hospital, regardless of the presence LUTS. Male patients aged over 18 years who were diagnosed as having MS, had not had an attack in the last 3 months, and were able to complete the questionnaires used in the study, were included. Patients with an indwelling urethral catheter, those who emptied their bladder with intermittent catheterization were not included in the study. Patients were excluded from the study if they were using medications that could affect bladder function and potentially influence the study outcomes, such as antimuscarinic drugs, beta‐3 agonists, or alpha‐adrenergic blockers for their existing LUTS. Patients who had LUTS before their MS diagnosis were not included in the study, as these symptoms may have been due to other causes, potentially affecting the results. The average age of the patients was 39, and 10 patients were over the age of 50. Patients were not subjected to ultrasonographic evaluation to rule out prostatic hypertrophy. They were only asked about whether they had a known history of prostatic hypertrophy based on their medical history. Demographic data, duration of MS, and disease type were recorded during the evaluations of the patients by a neurologist in the MS outpatient clinic. The severity of neurologic impairment was assessed by neurologists using the Kurtzke Expanded Disability Status Scale (EDSS, with scores ranging from 0 to 10) [10]. This widely used scale assesses impairment levels across eight functional systems, including pyramidal, cerebellar, brainstem, sensory, bowel and bladder, visual, cerebral, and others. For example, an EDSS score of 5 typically indicates that a person can walk without aid or rest for about 200 m, but experiences significant disability in other functional systems.

LUTS were evaluated using the International Incontinence Consultation Questionnaire‐Male LUTS questionnaire (ICIQ‐MLUTS). ICIQ‐MLUTS is a highly recommended questionnaire for evaluating LUTS in males and their impact on QoL, with the ability to individually assess voiding and storage symptoms, as well as continence status and their impact on patients' QoL [11]. ICIQ‐MLUTS consists of six storage symptoms, five emptying symptoms, and additional frequency and nocturia questions. The answers related to the storage and emptying symptoms are displayed as a 4‐point severity scale (0–4) including “never” (0), “occasionally” (1), “sometimes” (2), “most of the time” (3), and “all of the time” (4) and the degree of distress caused by the symptoms was evaluated on a scale of 0−10 using a Visual Analog Scale (VAS). Along with the number of daytime urination frequency and nocturia episodes, the degree of distress caused by these symptoms was also assessed using a VAS. The maximum scores for the voiding symptoms and incontinence symptoms subscales are 20 and 24, respectively [11].

The QoL of the patients was evaluated using the King's Health Questionnaire (KHQ) [12]. In this study, the first and second parts of the KHQ were used. The first part consists of two single‐item questions addressing general health perceptions and the impact of incontinence, and the second part includes questions evaluating role limitations, physical limitations, social limitations, personal relationships, emotions, sleep/energy, and severity measures. The minimum possible KHQ score is 0 (best health) and the maximum possible score is 100 (worst health).

Depression and anxiety were assessed using the Hospital Anxiety and Depression Scale, which includes HAD‐D and HAD‐A subscales. HAD scale consists of 14 items, 7 of which are depression symptoms and 7 are anxiety symptoms. Each item is scored from 0 (no distress) to 3 (maximum distress), where higher scores reflect a higher risk of depression or anxiety [13].

The medical research ethics committee of Ege University Medical Faculty approved the research proposal (22−12T/54). All participants were informed about the research and completed informed consent forms.

### 2.1 Statistical Analysis

The sample size calculation was based on expected LUTS prevalence of 70%, as reported in prior studies [6], to achieve a 95% confidence interval with a precision of ±10%, resulting in a minimum sample size of 89 patients. Patients were categorized into three groups based on ICIQ‐MLUTS incontinence and voiding scores. The three groups were compared across several demographic and clinical variables, including age, disease duration, EDSS score, and scores on the HAD‐A, HAD‐D, and KHQ. Multiple comparisons were initially performed between the three groups using the Kruskal−Wallis test. For variables where a significant difference was found (*p* < 0.05), pairwise comparisons between the groups were then conducted using Dunn's test with Bonferroni correction. Spearman's correlation test was used to determine the correlations of ICIQ‐MLUTS questionnaire subgroups with age, disease duration, EDSS scores, HAD‐A, HAD‐D, and KHQ subgroups Statistical analysis was performed using the IBM SPSS Statistics for Windows, Version 25.0 software package (IBM Corp. Released 2017, Armonk, NY). Statistical significance was set at *p* < 0.05.

## 3 Results

In this study, 100 men were evaluated with a mean age of 39.3 ± 9.3 (min. 20 to max. 63) years and a mean disease duration of 9.3 ± 6.0 years. The majority (93%) had relapsing‐remitting MS. The median EDSS was 2, 65% of the patients had EDSS scores between 0 and 2.0, and 11% of the patients had an EDSS score of ≥ 5. The most common storage symptoms were urgency (76%) and urgency IU (48%). The most common voiding symptoms were feeling of incomplete emptying (59%), and hesitancy and intermittency (52%). Table 1 presents the frequency of the urinary symptoms and the degree of distress (VAS) experienced by those who had these symptoms occasionally or sometimes and those who experienced them most of the time or all the time. The frequency of daytime urination of the patients was 1−6 times per day in 58%, 7−8 times per day in 21%, 9−10 times in 13%, 11−12 times in 2%, and 13 or more times in 6%. The mean discomfort levels as measured using a VAS in individuals who urinated 1−8 times per day and those who urinated ≥ 9 times per day were found as 1.4 ± 2.8 and 5.9 ± 3.4, respectively. The number of urinations during the night was 0 in 40% of the patients, one in 39%, two in 15%, three in 4%, and four or more in 2%. The mean discomfort levels measured using VAS in individuals who urinated 0−1 times at night (79%) and those who urinated ≥ 2 times at night (21%) were found as 0.8 ± 1.9 and 6.1 ± 3.5, respectively. The mean ICIQ‐MLUTS questionnaire incontinence and voiding scores were 4.2 ± 4.5 and 5.1 ± 5.3, respectively. ICIQ‐MLUTS questionnaire voiding and incontinence scores were statistically significantly correlated with disease duration, EDSS score, HAD‐A, HAD‐D scores, and all KHQ subgroups, except general health perceptions. ICIQ‐MLUTS questionnaire frequency and nocturia scores were statistically significantly correlated with HAD‐A, HAD‐D scores, and all KHQ subgroups, except general health perceptions and personal relationships (Table 2). ICIQ‐MLUTS questionnaire incontinence scores and voiding scores were grouped as those who answered 0 to all components (group 1), those who answered 1 or 2 (group 2), and those who answered 3 or 4 (group 3) according to the 4‐point severity scale (0–4). The comparison of these three groups in terms of age, disease duration, EDSS score, HAD‐A, HAD‐D, and KHQ components can be seen in Tables 3 and 4. KHQ and HAD‐A and HAD‐D scores were highest in group 3.

**Table 1:** Frequency of urinary symptoms and the degree of distress (VAS) suffered by those who experience these symptoms occasionally or sometimes and those who experience them most of the time or all of the time.

	Yes/no (%)	Occasionally/sometimes (%)	Bothering (VAS) mean ± SD	Most of the time/all of the time (%)	Bothering (VAS) mean ± SD
Hesitancy	57/43	42	3.2 ± 2.6	15	8.1 ± 1.9
Straining to continue urination	43/57	29	2.9 ± 2.5	14	8.5 ± 2.7
Decreased stream	41/59	32	3.4 ± 2.1	9	8.7 ± 1.6
Intermittency	52/48	35	2.3 ± 2.2	17	8.5 ± 1.7
Incomplete emptying	59/41	39	3.1 ± 2.8	20	7.5 ± 3.7
Urgency	76/24	48	3.2 ± 2.9	28	8.4 ± 2.3
Urgency urinary incontinence	48/52	38	4.8 ± 3.6	10	8.7 ± 3.1
Stress urinary incontinence	16/84	14	4.1 ± 3.1	2	10 ± 0
Unexplained urinary incontinence	14/86	8	3.6 ± 3.5	6	9.5 ± 0.8
Nocturnal enuresis	11/89	6	5.7 ± 3.9	5	10 ± 0
Post micturition dribble	46/54	37	4.2 ± 3.5	9	9.0 ± 1.7

**Table 2:** Correlations of ICIQ‐MLUT questionnaire subgroups with age, disease duration, EDSS score, HAD‐A, HAD‐D, and KHQ subgroups.

	ICIQ‐MLUTS questionnaire voiding score	ICIQ‐MLUTS questionnaire incontinence score	ICIQ‐MLUTS questionnaire frequency	ICIQ‐MLUTS questionnaire nocturia
Age	0.084	0.142	−0.086	0.039
Disease duration	0.312**	0.296**	0.182	0.132
EDSS score	0.367**	0.416**	0.014	0.026
HAD‐anxiety	0.521**	0.508**	0.257**	0.302**
HAD‐depression	0.483**	0.426**	0.225*	0.298**
KHQ‐general health perceptions	0.177	0.166	0.010	0.161
KHQ‐incontinence impact	0.728**	0.730**	0.391**	0.382**
KHQ‐role limitations	0.670**	0.678**	0433**	0.291**
KHQ‐physical limitations	0.710**	0.753**	0.385**	0.327**
KHQ‐social limitations	0.679**	0.654**	0.302**	0.258**
KHQ‐personal relationships	0.519**	0.549**	0.159	0.071
KHQ‐	0.601**	0.598**	0.267**	0.233*
KHQ‐Sleep/energy	0.635**	0.599**	0.313**	0.364**
KHQ‐Severity measures	0.563**	0.631**	0.311**	0.277**

** Spearman's rho correlation is significant at the 0.01 level (2‐tailed);

* Spearman's rho correlation is significant at the 0.05 level (2‐tailed).

**Table 3:** Comparison of the patients in terms of age, disease duration, EDSS score, HAD‐A, HAD‐D, and KHQ components according to the ICIQ‐MLUTS questionnaire incontinence scores.

	ICIQ‐MLUTS questionnaire incontinence score		
	Group‐1 0 (*n* = 21)	Group‐2 1/2 (*n* = 49)	Group‐3 3/4 (*n* = 30)
Age	36.4 ± 7.9	40.4 ± 10.1	39.5 ± 8.5
Disease duration	7.1 ± 4.9	9.9 ± 6.5	9.7 ± 5.8
EDSS score	1.0 ± 1.2*	2.4 ± 1.9	2.5 ± 1.9*
HAD‐anxiety	4.5 ± 2.9	5.8 ± 3.4**	8.4 ± 4.3*
HAD‐depression	5.0 ± 3.5	5.4 ± 3.5*	8.3 ± 4.4*
KHQ‐health perception	18.4 ± 22.1	17.7 ± 21.6	21.4 ± 26.3
KHQ‐ Incontinence effect	11.5 ± 18.9	15.8 ± 25.9	38.1 ± 42.9
KHQ‐role limitation	4.0 ± 9.8	8.9 ± 16.5***	29.6 ± 34.4***
KHQ‐physical limitation	3.2 ± 7.7	9.9 ± 16.7***	33.1 ± 37.6***
KHQ‐social limitation	2.2 ± 4.1	8.1 ± 17.8**	18.9 ± 25.3***
KHQ‐personal relationships	1.0 ± 1.4	8.1 ± 17.7	12.8 ± 23.3**
KHQ‐emotions	7.1 ± 14.3	6.6 ± 10.4**	22.2 ± 24.8***
KHQ‐sleep/energy	4.0 ± 8.2	6.8 ± 11.4	18.7 ± 26.5
KHQ‐symptom severity	10.1 ± 15.8	9.0 ± 10.5**	22.8 ± 21.8***

*Note:* Comparison of Group‐1 and 2 is marked in the first column; Comparison of Group‐2 and 3 is marked in the second column; Comparison of Group‐1 and 3 is marked in the third column; Kruskal−Wallis test.

* *p* < 0.05;

** *p* < 0.01;

*** *p* < 0.001.

**Table 4:** Comparison of the patients in terms of age, disease duration, EDSS score, HAD‐A, HAD‐D, and KHQ components according to the ICIQ‐MLUTS questionnaire voiding scores.

	ICIQ‐MLUTS questionnaire voiding score		
	Group‐1 0 (*n* = 21)	Group‐2 1/2 (*n* = 52)	Group‐3 3/4 (*n* = 27)
Age	41.0 ± 9.3	38.6 ± 9.2	39.4 ± 9.4
Disease duration	8.1 ± 4.9	8.2 ± 5.9*	12.2 ± 7.3
EDSS score	1.7 ± 1.7	1.9 ± 1.6	3.0 ± 2.1
HAD‐anxiety	4.4 ± 3.5	5.5 ± 3.1**	9.4 ± 3.6***
HAD‐depression	4.8 ± 3.7	5.5 ± 3.4**	9.2 ± 4.3***
KHQ‐health perception	16.1 ± 22.5	17.2 ± 21.2**	24.5 ± 26.5***
KHQ‐incontinence effect	5.2 ± 11.8**	19.4 ± 29.3**	38.4 ± 41.4***
KHQ‐role limitation	0.8 ± 1.0**	11.7 ± 20.7**	28.9 ± 32.9***
KHQ‐physical limitation	2.4 ± 7.1**	11.8 ± 19.7*	32.6 ± 37.6***
KHQ‐social limitation	0.8 ± 1.0	8.7 ± 16.5	20.1 ± 27.3**
KHQ‐personal relationships	1.7 ± 3.7	6.6 ± 14.5**	15.7 ± 27.1***
KHQ‐emotions	4.5 ± 9.7*	7.4 ± 11.8**	24.2 ± 25.5***
KHQ‐sleep/energy	2.4 ± 7.1*	7.2 ± 11.7	20.5 ± 26.9**
KHQ‐symptom severity	5.9 ± 9.2	12.3 ± 13.7***	21.4 ± 22.9***

*Note:* Comparison of Group‐1 and 2 is marked in the first column; Comparison of Group‐2 and 3 is marked in the second column; Comparison of Group‐1 and 3 is marked in the third column; Kruskal−Wallis test.

* *p* < 0.05;

** *p* < 0.01;

*** *p* < 0.001.

## 4 Discussion

This cross‐sectional study investigated the frequency and distress of LUTS in men with MS, and their correlation with QoL, depression, and anxiety. Data on LUTS in men with MS are obtained from studies in which male and female patients were recruited together, and as expected, the percentage of male patients was low. The prevalence and severity of UI in patients with MS were investigated in a study performed by Zeccaa et al. who found a positive correlation between International Consultation on Incontinence Questionnaire scores and female sex indicating that incontinence affected QoL more in women [7]. On the other hand, in another study, male and female patients reported similar urinary symptom scores [6]. The only study that included only male patients with MS, conducted by Tome et al., evaluated the prevalence and relationship between LUTS and sexual dysfunction. Due to the lack of information on this topic in the literature, we included only male patients in our study and aimed to highlight the importance of the situation in male patients with MS.

Tome et al. reported that 97.6% of their patients experienced LUTS, predominantly storage symptoms (85%), including urgency (70.7%), frequency (46.3%), and urgency IU (36.6%). Voiding symptoms were reported by 72.5% of patients, notably hesitancy (70.7%), intermittency (46.3%), and incomplete emptying sensation [9]. They defined increased urination frequency as ≤ 2 h intervals (46.3%). In our study, the frequency of daytime urination in the ICIQ‐MLUTS questionnaire were grouped in five categories (1−6 times, 7−8 times, 9−10 times, 11−12 times, and 13 or more times per day). Frequency is generally considered to be ≥ 8 times during the day, but according to the categorization in the ICIQ‐MLUTS, we could not assess this value. Twenty‐one percent of patients had a frequency of ≥ 9, and 42% had a frequency of ≥ 7.

The ICIQ‐MLUTS used in our study is derived from the International Continence Society for males (ICSmSF) questionnaire used in the study by Tome et al. Compared with the patients in Tome et al.'s study, our patients had lower median EDSS scores (2 vs. 3), slightly shorter mean disease duration (9.3 ± 6.0 vs. 10.5 ± 7.3 years), and similar mean ages (39.3 ± 9.3 vs. 40.7 ± 10.1 years) [9]. In our study, the most common clinical type of MS was relapsing‐remitting MS (93%). However, no information was provided about the type of MS in the study by Tome et al. In their study, 39% of the patients had an EDSS score of ≥ 5, whereas in our study, this rate was 11% [9]. The differing proportions of patients with more advanced neurologic disability and MS type may have affected the results. Zeccaa et al. found that UI had a strong positive correlation with EDSS scores in both sexes [7]. Araki et al. showed that storage but not voiding symptoms correlated with EDSS scores [3]. Tome et al.'s findings were consistent with these results, confirming the association between neurologic impairment and storage symptoms. In our study, unlike these results, both storage and voiding symptoms were found to be correlated with EDSS scores.

A longitudinal study reported that the proportion of patients with MS with at least one bladder dysfunction symptom had significantly increased over time among both men and women. At any follow‐up point, there was a significant association with high levels of physical disability and health‐related QoL for both sexes [14]. Quarto et al. showed that urinary symptoms impaired several areas of QoL ranging from physical activities, sleep, and energy to emotions and relationships in patients with MS [15]. In our study, ICIQ‐MLUTS questionnaire scores for voiding and incontinence were found to be significantly correlated with all KHQ subgroups except general health perceptions, and scores for frequency and nocturia were significantly correlated with all KHQ subgroups except general health perceptions and personal relationships.

In the study performed by Tome et al., LUTS affected the QoL of male patients mildly in 43.9%, moderately in 9.8%, and severely in 19.5% [9]. Khalaf et al. conducted a cross‐sectional online survey of 1048 patients with MS (81% female) and found that urgency IU (52.6%) was the most prevalent LUTS. Both urgency and urgency incontinence significantly decreased QoL [16]. Nazari et al. studied the QoL of 602 patients with MS with voiding dysfunction, 21.3% of whom were men [4]. They used the International Prostate Symptom Score (IPSS) for LUTS assessment and the MSQOL‐54 to evaluate QoL. Mixed, storage, and voiding symptoms were present in 51.1%, 28.1%, and 4.5% of women, and 56.5%, 16.3%, and 10.1% of men, respectively. About 15.4% of women and 17.1% of men had no urinary symptoms. Patients with MS with LUTS had lower QoL scores in all dimensions except health changes, with significant differences in physical and mental health dimensions compared with those without LUTS. Ziadeh et al. found that in patients with persistent LUTS, storage symptoms were more bothersome than voiding symptoms and had a greater negative impact on QoL [17]. Unlike the Urinary Bothersome Questionnaire in MS, which uses single questions to asess bother from storage and voiding symptoms in patients with MS [18], our study provides a more granular assessments. We used a VAS to evaluate the bother associated with each individual LUTS.

Depression and anxiety are common in patients with MS. A systematic review and meta‐analysis encompassing 58 articles and 87,756 patients with MS reported pooled mean prevalences of 30.5% for depression and 22.1% for anxiety [19]. Vrijens et al. found a positive association between depression and overactive bladder (OAB) in 26 studies and between anxiety and OAB in six studies. The results of this systematic review showed that there was abundant and quality evidence of a positive association between the co‐occurrence of OAB and depression, and to a lesser extent, OAB and anxiety. The association between OAB and depressive symptoms may be bidirectional. Evidence for the association between OAB and anxiety is too poor to make meaningful statements [20]. One possible explanation for the association between affective disorders and OAB is that UI can cause social and functional impairment, leading to stress, and subsequently, depression [21]. Both syndromes may share common biologic pathways [22].

The relationship between LUTS and depression and anxiety in MS has been investigated in very few studies. Khalaf et al. showed that patients with MS with urinary urgency or urgency UI were significantly more likely to report experiencing depression compared with those with no/minimal urinary symptoms [16]. Tudor et al. showed that patients with MS with depression had more pronounced LUTS. Positive correlations were found between depression and LUTS on UI short‐form and LUTS related QoL [23]. In the study conducted by Akkoc et al. on women with MS, a moderate positive correlation was found between OAB‐V8 scores and HAD‐A and HAD‐D scores [24]. In the recent study, the ICIQ‐MLUTS questionnaire scores for voiding, incontinence, frequency, and nocturia were found to be significantly correlated with HAD‐A and HAD‐D scores. The ICIQ‐MLUTS questionnaire incontinence scores and voiding scores were grouped as those who answered 0 to all components (group 1), those who answered 1 or 2 (group 2), and those who answered 3 or 4 (group 3) according to the 4‐point severity scale (0–4). The HAD‐A and HAD‐D scores were found to be highest in group 3. These results show that depression and anxiety are higher in those with severe LUTS.

To the best of our knowledge, this is the first study to use the ICIQ‐MLUTS questionnaire in patients with MS and to evaluate the correlation between LUTS and QoL, depression, and anxiety. The primary advantage of the ICIQ‐MLUTS over previous questionnaires is its ability to evaluate voiding and storage symptoms, as well as continence status, and their impact on patient QoL individually. This unique feature allows for more detailed discussions of treatment alternatives with patients. The strength of our study lies in its comprehensive assessment of LUTS, QoL, depression, and anxiety among male patients with MS within a clinical framework, thus avoiding solely relying on self‐reported data or telephone interviews. Our study has some limitations. We cannot generalize the results to all types of MS because most patients had RRMS. As a methodological limitation, the exclusion of patients with pre‐existing LUTS may not entirely eliminate non‐MS‐related causes, and the LUTS observed in MS may arise from overlapping or interacting pathophysiological mechanisms. Another possible limitation of the present study is that the use of antidepressant medication was not considered when investigating the depression status of the patients. Moreover prostate ultrasonographic evaluation was not performed in the study; only the presence of known prostate hypertrophy was inquired through medical history. Although the statistical analyses we conducted appropriate with the purpose of our study, future research employing multivariate approaches with larger sample sizes may be beneficial for examining these relationships in greater detail.

## 5 Conclusion

LUTS were common and distressing in men with MS and were significantly associated with reduced QoL, and increased anxiety and depression levels. However, further studies are needed to determine the causal direction and independent contribution of LUTS to psychological outcomes.

## Ethics Statement

The medical research ethics committee of Ege University Medical Faculty approved the research proposal (Number: 22‐12 T/54).

## Consent

All the participants were informed about the research and completed the informed consent form.

## Conflicts of Interest

The authors declare no conflicts of interest.

## Permission to Reproduce Material From Other Sources

Permission to use clinical records was obtained from the chair of the department.

## Data Availability

The data can be accessed upon request from the corresponding author.
